# Case report of transplant renal artery stenosis secondary to mechanical renal artery kinking: Balloon angioplasty as a supportive diagnostic tool?

**DOI:** 10.1016/j.ijscr.2021.106052

**Published:** 2021-05-29

**Authors:** Brooklyn L. DeVries, Brendan Wechsler, Douglas Yim

**Affiliations:** a USD Sanford School of Medicine, 1400 W. 22nd St., Sioux Falls, SD 57105, United States of America; b Avera Mckennan Hospital, University Health Center Department of Interventional, Radiology, 1325 S. Cliff Avenue, Sioux Falls, SD 57105, United States of America

**Keywords:** Transplant renal artery stenosis, Renal artery kinking, Balloon angioplasty, Renal angiogram, Case report

## Abstract

**Introduction and importance:**

Renal vascular complications are a significant cause of morbidity in living donor renal transplantation. Among renal vascular complications, transplant renal artery stenosis (TRAS) secondary to mechanical kinking is a rare but important cause of early graft dysfunction. Identifying this phenomenon and correcting the underlying cause is critical to graft viability in the post-operative period. This case illustrates the importance of balloon angioplasty in identifying this complication and prompting surgical correction.

**Case presentation:**

We describe the case of a 67-year-old male who received a right-sided living donor kidney graft for Stage IV Chronic Kidney Disease secondary to biopsy proven Ig-A nephropathy. In the post-operative course, serum creatinine remained elevated and Doppler showed low-normal vascular flow velocities. Renal angiogram indicated transplant renal artery stenosis secondary to the rare phenomenon of mechanical kinking. Findings noted during unsuccessful angioplasty supported the diagnosis and surgical repositioning of the graft provided definitive repair. Post-operative serum creatine trended down and urine output improved within 24 h. Patient was stable at two month follow up.

**Clinical discussion:**

Transplant renal artery stenosis secondary to mechanical kinking can cause significant graft dysfunction in the post operative period. Previous case reports and literature review has found balloon angioplasty to be ineffective in correcting this underlying cause of TRAS. In line with previous reports, balloon angioplasty failed to correct the stenosis; however, this provided additional diagnostic information by identifying the kink and prompting surgical repair.

**Conclusion:**

Transplant renal artery stenosis secondary to mechanical kinking can be difficult to identify by renal angiogram alone. Attempted balloon angioplasty can confirm the diagnosis and prompt definitive surgical repair.

## Highlights

• Rare case of transplant renal artery stenosis secondary to renal artery kinking
• Balloon angioplasty aided in distinguishing between TRAS and TRAS secondary to mechanical kinking.
• Early identification through renal angiogram and unsuccessful angioplasty permitted successful surgical repair.

## 1 Introduction

Renal vascular complications are a significant cause of morbidity in living donor renal transplantation. These complications include hemorrhage, renal artery thrombosis, renal vein thrombosis, aneurysms/fistulas, and post-transplant renal artery stenosis [1]. Among these causes of post-transplant renal vascular complications, transplant renal artery stenosis (TRAS) stands out as one of the most common complications associated with increased graft loss and morbidity [2]. TRAS is often caused by surgical complications related to the creation of the anastomosis, immune modulating vascular injury, intimal hyperplasia, atherosclerotic disease, and in rare cases mechanical renal artery kinking [3]. Several hypotheses to the cause of TRAS secondary to renal artery kinking have been suggested including restricted space within the transplant bed in the iliac fossa, longer right renal artery compared to the right renal vein, or post-operative shifting of graft/abdominal contents [4,5,6]. These underlying factors are thought to cause malposition of the graft making it prone to kinking but the rarity of this phenomenon leaves much to be determined. Previous case reports identified only thirteen instances of post-transplant renal artery kinking between 1994 and 2019 [4,[7], [8], [9], [10], [11], [12], [13]]. Due to the rarity of this post-operative complication, a true incidence is unknown. In one retrospective study of vascular complications at a single center, seven instances of post-transplant renal artery kinking were identified which represented 0.3% of post-operative renal transplant complications [13]. Here we describe a case of post-transplant renal artery kinking suspected based on renal angiogram findings and supported by unsuccessful balloon angioplasty attempts that required surgical correction with graft repositioning for definitive repair.

This case report has been reported in line with SCARE Criteria [14].

## 2 Case report

**Fig. 1:**
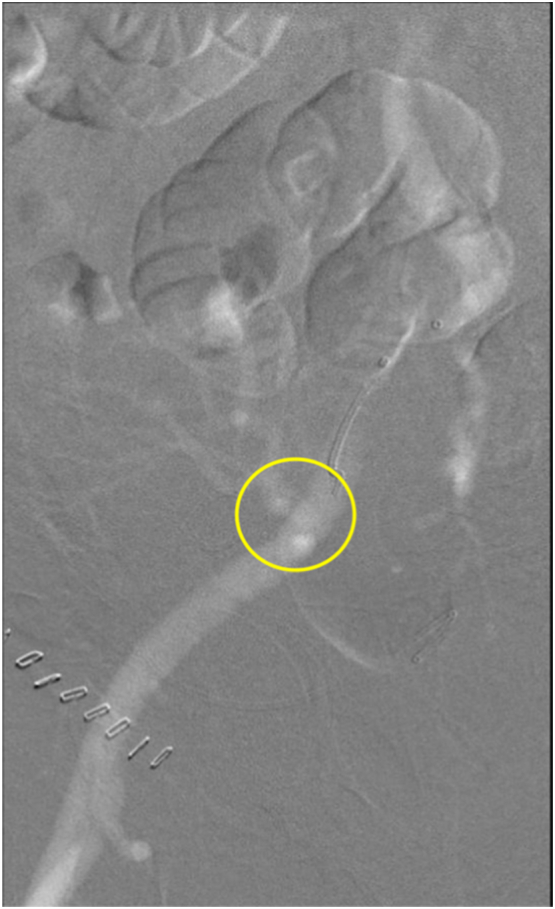
Renal CO2 angiogram. Renal artery stenosis likely due to kinking is observed.

**Fig. 2:**
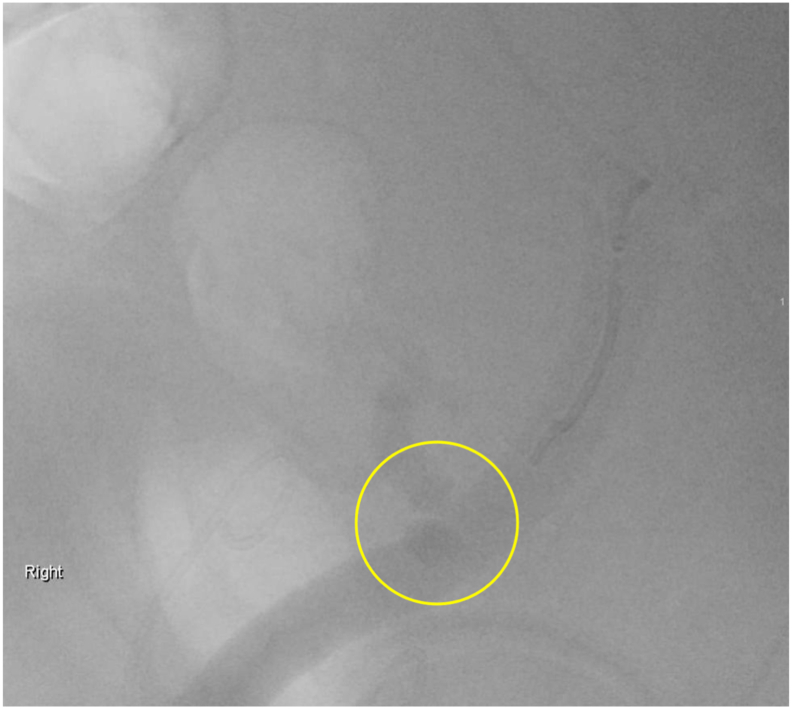
Renal angiogram with limited iodine contrast. Renal angiogram with contrast shows evidence of stenosis due to kinking.

**Fig. 3:**
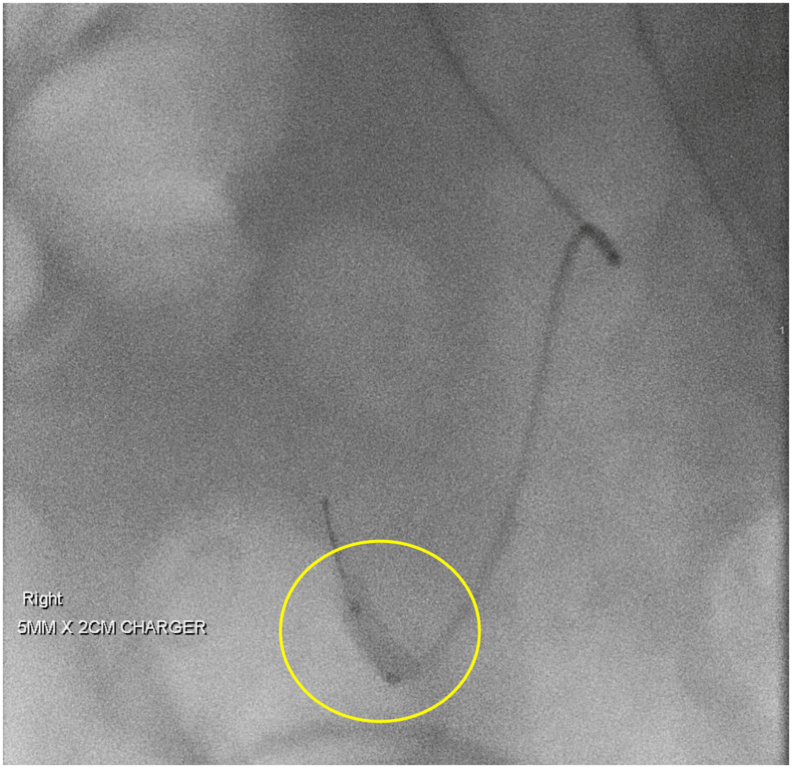
Balloon angioplasty of renal artery kink. Balloon fully dilated without resistance across stenotic region suspicious for kinking.

**Fig. 4:**
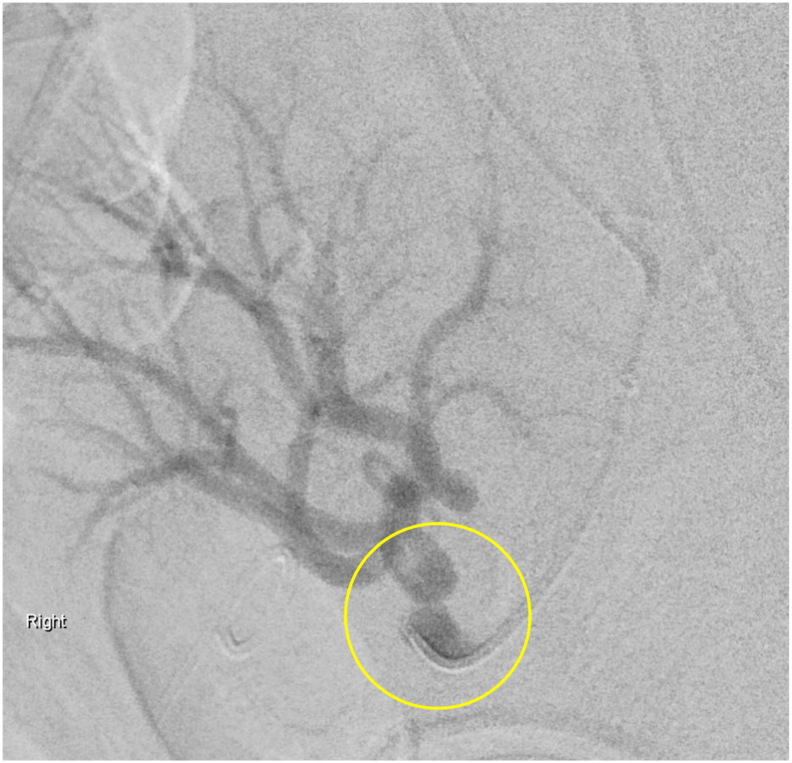
Post-balloon angioplast renal angiogram. Renal angiogram shows continued stenosis secondary to renal artery kinking after attempted angioplasty.

A 67-year-old male underwent a live, related renal artery transplant due to Stage IV Chronic Kidney Disease secondary to biopsy proven IgA nephropathy. Patient has a past medical history of Type II Diabetes Mellitus, HTN, OSA, bladder cancer s/p TURP, systolic heart failure with implanted defibrillator, dyslipidemia, and obesity with BMI of 38.0 kg/m^2^. He is a retired maintenance worker and never smoker with no alcohol use. The kidney of his biological brother was transplanted into the right iliac fossa with end to side anastomosis of graft renal vessels to the external iliac vessels. The surgical course was uneventful. Surgery was performed by a board certified transplant surgeon with over 10 years of experience at a regional community practice. Pre-operative creatinine was 3.3 mg/dl. Post-operative creatinine was 3.1 mg/dl and increased to 3.3 mg/dl on post-operative day one. Urine output was satisfactory at 220 ml/h. Immediate post-operative ultrasound showed no evidence of perinephric fluid collection and low-normal renal artery peak systolic velocity measures of 41 cm/s at the hilum, 46 cm/s at the mid-portion, and 57 cm/s at the anastomosis with resistive indices of 0.6 at the upper, middle, and lower pole. On post-operative day one, repeat ultrasound revealed decreasing renal artery peak systolic velocities at the hilum (32 cm/s) and mid-portion (33 cm/s), but increased peak systolic velocity at the anastomosis (129 cm/s) with resistive indices of 0.73 at the upper, 0.63 at the middle, and. 0.60 at the lower pole. Follow-up renal nuclear medicine scan showed delayed perfusion to the renal transplant. Renal CO2 angiogram demonstrated evidence of kinking of the transplant's renal artery (Fig. 1). Renal angiogram with judicious contrast supported this finding as well (Fig. 2). Attempted balloon angioplasty revealed a lesion that did not behave like a true stenosis in that very little resistance was encountered during inflation with no stricture noted in the balloon (Fig. 3). Renal angiogram showed continued kinking after deflation of the balloon (Fig. 4). This evaluation and attempted angioplasty was performed by a board certified interventional radiologist with more than 15 years of experience at a regional community practice. Due to unsuccessful angioplasty and evidence of mechanical kinking on renal angiogram, the patient underwent surgical evaluation, and a renal artery kink was identified 1.5 cm distal to the anastomosis. The graft was repositioned with upper pole placed in the posterior pelvis and lower pole positioned towards the incision to avoid kinking of the vasculature. The operative course was uneventful. Post-operative ultrasound revealed increasing peak renal artery systolic velocities at the hilum (268 cm/s, high-resistance wave form), mid-portion (128 cm/s), and anastomosis (173 cm/s, high-resistance wave form) with resistive indices of 0.51 at the upper, 0.58 at the middle, and 0.60 at the lower pole. Serum creatinine decreased to 2.8 mg/dl on post-operative day one and 2.1 mg/dl on post-operative day two. Urine output remained strong at 2.5l in 24 h post-op indicating successful repositioning. The patient developed a seroma and borderline evidence of rejection on renal biopsy in the continued post-operative course. At two months follow up the patient is stable with a creatinine of 1.3 mg/dl.

## 3 Discussion

Mechanical renal artery kinking is a rare cause of TRAS and an important complication of renal transplantation that can lead to early graft dysfunction [[1], [2]]. Identification of TRAS by post-operative Doppler ultrasound screening and confirmation by digital subtraction angiography is the gold standard in diagnosing this complication [3]. In this case, persistently elevated creatinine and low-normal renal artery velocities on Doppler ultrasound led to an investigation of graft perfusion by renal angiogram that identified TRAS. Furthermore, attempted balloon angioplasty revealed a lesion that did not behave like a stenosis in that very little resistance was encountered during inflation with no stricture noted in the balloon. This unsuccessful balloon angioplasty further supported kinking as the etiology for early graft dysfunction. These results were able to guide management towards definitive surgical repair.

Early recognition and correction of this complication is paramount to preventing graft loss and morbidity, but management of this complication is not currently well defined. Several management techniques have been successfully utilized including conservative management [5], endovascular repair [8,10], or surgical correction [7,9,11,12]. In cases where the renal artery kink is near the anastomosis and there is evidence of early graft dysfunction (rising creatinine, decreased urine output, or uncontrolled hypertension), the renal artery kinks are often resistant to endovascular repair and require prompt surgical correction with nephropexy or re-anastomosis [3,7,9,10,11,12]. It is likely the kink does not act as a true stenosis and frequently rebounds to its former configuration after percutaneous transluminal angioplasty or is propagated distally during stenting [3]. With malposition of the graft the most likely cause of kinking, it can be deduced that surgical re-anastomosis or repositioning of the graft is often required to permanently correct the dysfunction. Based on literature review, only 15% (2/13) of TRAS secondary to mechanical artery kinking were successfully treated with endovascular repair [4,[8], [9], [10], [11], [12], [13]]. While our case was also resistant to endovascular repair, we suggest balloon angioplasty as an important diagnostic clue in the identification of TRAS secondary to mechanical artery kinking. Mechanical artery kinking can be difficult to identify on angiogram alone due to the tortuous nature of renal vessels. Suggestive renal angiogram in combination with notable features seen during unsuccessful balloon angioplasty can provide higher confidence in diagnosis and stronger recommendation for definitive surgical repair when managing TRAS secondary to mechanical artery kinks.

## 4 Conclusion

Post-transplant renal artery kinking is a very rare complication with few cases reported in the literature. This report describes an additional case of post-transplant renal artery kinking identified by renal angiogram and unsuccessful angioplasty attempts that was managed surgically with graft repositioning. In this case, failed angioplasty provided valuable supporting evidence that mechanical artery kinking was the cause of graft dysfunction. With the addition of this case, it is becoming clearer that balloon angioplasty may be a supporting diagnostic tool in identifying arterial kinking and that surgical correction is the most definitive management for TRAS secondary to renal artery kinks. Due to the low incidence of this transplant complication, further research is required to determine the true incidence and definitive management protocols.

## Provenance and peer review

Not commissioned, externally peer-reviewed.

## Source of funding

None.

## Ethical approval

Received ethics exemption per Avera Mckennan Hospital and University Health Center IRB Policy for case reports. Avera IRB Personnel. Reference Number: 2021.036.

## Consent

Written informed consent was obtained from the patient for publication of this case report and accompanying images. A copy of the written consent is available for review by the Editor-in-Chief of this journal on request.

## Registration of research studies

Not applicable.

## Guarantor

Brooklyn DeVries.

## CRediT authorship contribution statement

Brooklyn Devries: Literature Review, Original Draft, Corresponding Author.

Brendan Wechsler: Review and editing.

Dr. Douglas Yim: Conceptualization, Review and Editing.

## Declaration of competing interest

The authors report no competing interests.
